# Validation of Single Nucleotide Variant Assays for Human Leukocyte Antigen Haplotypes HLA-B*15:02 and HLA-A*31:01 Across Diverse Ancestral Backgrounds

**DOI:** 10.3389/fphar.2021.713178

**Published:** 2021-07-26

**Authors:** Amanda Buchner, Xiuying Hu, Katherine J. Aitchison

**Affiliations:** ^1^ Department of Psychiatry, Faculty of Medicine and Dentistry, University of Alberta, Edmonton, AB, Canada; ^2^ Neuroscience and Mental Health Institute, University of Alberta, Edmonton, AB, Canada; ^3^ Department of Medical Genetics, Faculty of Medicine and Dentistry, University of Alberta, Edmonton, AB, Canada

**Keywords:** HLA antigens, adverse drug reactions, carbamazepine, psychiatry, pharmacogenetics, precision medicine, single nucleotide variants, oxcarbazepine

## Abstract

The human leukocyte antigen haplotypes HLA-B*15:02 and HLA-A*31:01 have been linked to life-threatening adverse drug reactions to the anticonvulsants carbamazepine and oxcarbazepine. Identification of these haplotypes *via* pharmacogenetic techniques facilitates implementation of precision medicine to prevent such reactions. Using reference samples from diverse ancestral origins, we investigated the test analytical validity (i.e., ability to detect whether or not the haplotypes were present or absent) of TaqMan assays for single nucleotide variants previously identified as potentially being able to “tag” these haplotypes. A TaqMan custom assay for rs10484555 and an inventoried assay for rs17179220 and were able to identify with 100% sensitivity and 100% specificity HLA-B*15:02 and HLA-A*31:01 respectively. A custom assay for rs144012689 that takes into account a neighboring single nucleotide variant with manual calling was also able to identify HLA-B*15:02 with 100% sensitivity and 100% specificity. A custom assay for rs1061235 identified HLA-A*31:01 with 100% sensitivity and 95% specificity. The slight reduction in specificity for the latter was owing to another haplotype (HLA-A*33:03) also being detected. While any positive call using the rs1061235 assay could therefore be further investigated, as the presence of the HLA-A*31:01 haplotype confers adverse drug reaction risk, the absence of false negatives (indexed by sensitivity) is more important than false positives. In summary, we present validated TaqMan assay methodology for efficient detection of HLA haplotypes HLA-B*15:02 and HLA-A*31:01. Our data are relevant for other genotyping technologies that identify, or have the potential to identify, these haplotypes using single nucleotide variants.

## Introduction

Adverse drug reactions (ADRs) pose a significant burden to the healthcare system, comprising a significant portion of hospital admissions, mortality, and overall healthcare costs (Baker et al., 2004; Pirmohamed et al., 2004). Globally, the cost of drug-related morbidity and mortality in 2000 was estimated at $177.4 billion, a figure that nearly doubled the previous 1995 estimate (Ernst and Grizzle, 2001). In addition to the strictly economical cost, the distribution of ADRs across the population highlights disparities within the healthcare system. In a United States study, it was found that older individuals, men, Black persons, and individuals residing in extremely rural areas (counties with less than 10,000 residents) experienced higher mortality associated with ADRs (Shepherd et al., 2012). This indicates the need for action to prevent ADRs to ensure that subsets of our population are not facing disproportionate challenges in their access to and quality of care.

Pharmacogenomics provides the opportunity to prevent ADRs. *HLA-A* and *HLA-B* are genes encoding human leukocyte antigens (HLAs), part of the class I major histocompatibility complex (MHC) (Wieczorek et al., 2017). This complex is a group of cell surface proteins involved in the presentation of antigenic peptides to T cells to induce an immune response (Wieczorek et al., 2017). The region that encodes the HLA complex consists of ∼260 genes in a ∼4-Mb span on chromosomal region 6p21.3, base pair positions 29,640,000–33,120,000 from the Genome Reference Consortium Human Build 37, hg1 (Trowsdale and Knight, 2013). The most polymorphic HLA class I and class II proteins are each expressed from three gene regions (MHC class I: HLA-A, -B, -C; MHC class II: (HLA-DR, -DP, -DQ), which are all highly polymorphic (Trowsdale and Knight, 2013). The HLA complex is the most polymorphic human genetic system, with over 8000 functional class I variants (Choo, 2007; Illing et al., 2017). Variations in the HLA genes play an important role in determining susceptibility to infection and to autoimmune disease, and can contribute to the recognition of drugs and their metabolites as foreign antigens by T-cell receptors with a consequent immunological reaction (Fan et al., 2017).

Specifically, the HLA haplotypes HLA-B*15:02 and HLA-A*31:01 are predictive of severe cutaneous reactions to the anticonvulsant drug carbamazepine, also prescribed as a mood stabilizer in psychiatry (Fan et al., 2017; Gui et al., 2018; Carvalho Henriques et al., 2020c). Cutaneous ADRs are one of the most common types of ADRs, typically occur in the first two to three months of drug use, and can range from a mild skin rash to a life-threatening reaction such as Stevens-Johnson syndrome and the more severe form of this, known as toxic epidermal necrolysis (SJS/TEN). SJS/TEN is characterized by rapid blistering, cutaneous and muscle detachment, and has a mortality rate of 10–30% (Fan et al., 2017; Mullan et al., 2019). Performing testing for these haplotypes can therefore be potentially life saving, with a Hong Kong study finding that testing for HLA-B*15:02 reduced the incidence of SJS/TEN induced by carbamazepine from 0.24 to 0% (Chen, Liew, and Kwan, 2014). The HLA-A*31:01 haplotype has a frequency of 2–5% in Northern European populations, with its presence increasing the risk of carbamazepine-induced SJS/TEN from 5 to 26% (Mccormack et al., 2011). As a result, before prescribing carbamazepine, it is advised that a genetic test be performed to ensure the patient does not carry either of these risk haplotypes (Amstutz et al., 2014).

Although genetic testing can greatly improve patient care and reduce the potential for ADRs, the uptake of screening has been limited because of the technical and economic barriers associated with conventional HLA haplotyping methods. Due to the highly polymorphic nature of the complex, the “gold-standard” for HLA haplotyping is sequencing-based methods and other specialized techniques, such as polymerase chain reaction-based sequence-specific priming and microbead hybridization (Dunckley, 2012; Lazaro et al., 2013; Edgerly and Weimer, 2018). However, these strategies are time consuming and expensive; the mean cost for testing in North America is $363.65 USD (Verbelen, Weale, and Lewis, 2017). This may limit the availability of such testing in patient groups in which it should be offered. Current United States Food and Drug Administration (FDA) recommendations for carbamazepine state that “prior to initiating carbamazepine therapy, testing for HLA-B*1502 should be performed in patients with ancestry in populations in which HLA-B*1502 may be present” and that “HLA-B*1502 is found almost exclusively in patients with ancestry across broad areas of Asia” (US National Library of Medicine, 2021). As a result, in many jurisdictions, HLA-B*15:02 testing for hypersensitivity to carbamazepine is offered only to Asians (Fang et al., 2019; Alberta Precision Laboratories, 2021; US National Library of Medicine, 2021).

SNV based methods of genotyping permit high throughput economical typing (Erlichster, Goudey, Skafidas, and Kwan, 2019) and therefore using such to “tag” the HLA haplotypes offers a potential route of making the identification of the at risk haplotypes more widely available (Petersdorf and O’hUigin, 2019). While previous studies had identified potential haplotype tagging SNVs for HLA-B*15:02 and HLA-A*31:01, there is no consensus regarding which SNVs can be used across ancestral groups. In fact, some of the literature is contradictory. For example rs2844682 and rs3909184 have been previously suggested as tag SNVs for HLA-B*15:02 (de Bakker et al., 2006; He et al., 2015), while another study concluded that these SNVs had no clinical value for identifying HLA-B*15:02 carriers (Zhu et al., 2015). In addition, many studies have restricted their populations to individuals from a particular ethnic group (de Bakker et al., 2006; Liu et al., 2015; Maekawa et al., 2015; Zhou et al., 2016; Gui et al., 2018). Further, methodologies vary greatly between studies, with some estimating validity based on databases (Erlichster et al., 2019) and others using genotyping (He et al., 2015; Fang et al., 2019).

A haplotype-tagging SNV method of screening US patients for HLA-B*15:02 without ethnicity-based preselection was able identify more than twice the number of carriers at risk of a carbamazepine-related ADRs than screening patients of Asian ancestry alone (Fang et al., 2019). This supports a prior suggestion that the FDA recommendations be revised to capture the broader range of patients who may be at risk for ADRs in response to carbamazepine (Payne, 2014). The aim of this study was therefore to generate validated SNV assays for the identification of HLA-B*15:02 and HLA-A*31:01 across a range of ancestral groups.

## Materials and Methods

### Selection of Potential Haplotype Tagging SNVs for Analytical Validation

A qualitative literature review was conducted to select four potential tagging SNVs for investigation across ancestral groups (Tables 1, 2). Criteria for candidate tag SNV selection included: recency of the publication, population size, ancestry, method of tag selection, and validation method. No studies were excluded. Two SNVs were selected for each haplotype so that the two could be tested in parallel to determine if one had greater sensitivity and specificity than the other for their respective haplotypes - as a method of confirming prior literature on each SNV whilst allowing for possible novel findings. SNVs rs10484555 and rs144012689 were chosen to tag HLA-B*15:02 and SNVs rs17179220 and rs1061235 were chosen to tag HLA-A*31:01.

**TABLE 1:** Prior data on HLA-A*31:01 potential tag SNVs.

SNVs	Population (size)	Tagging ability	Validation methodology	Publication(s)
rs17179220	Diverse (955)	100% sn, 99.6% sp	Tested against public database	Erlichster et al. (2019)
	Han Chinese (20635)	n/a	Tested on reference panels	Zhou et al., 2016
rs2571375	Han Chinese (184)	100% sn, 96.7% sp	Tested on reference panels	Gui et al. (2018)
rs1061235	Diverse (160)	100% sn, 84% sp	TaqMan SNP genotyping assay	He et al. (2015)
	Norwegian (204)	100% sn, 99.5% sp	PCR-restriction fragment length polymorphism (RFLP) assay	Thorstensen et al. (2014)
	Diverse (361)	n/a	Tested on reference panels	de Bakker et al. (2006)
rs1150738, rs3869066, rs259945	Japanese (210)	100% sn, 100% sp	PCR-RFLP assay	Maekawa et al. (2015)
rs41541222	Asian (466)	n/a	Sequence-specific primer (SSP) PCR	Aoki et al. (2012)

Sn = sensitivity, sp = specificity, n/a = information that is not available within the publication. Underlined SNVs were selected for technical validation.

**TABLE 2:** Prior data on HLA-B*15:02 potential tag SNVs.

SNVs	Population (size)	Tagging ability	Validation methodology	Publication(s)
rs10484555	Diverse (955)	100% sn, 98.7% sp	Tested against public database	Erlichster et al. (2019)
	Han Chinese (880)	100% sn, 99.3% sp	Sequencing	Liu et al. (2015)
rs144012689	United States (28897)	100% sn, 99.7% sp	TaqMan SNP genotyping assay	Fang et al. (2019)
	Han Chinese (184)	96.7% sn, 99.3% sp	Tested on reference panels	Gui et al. (2018)
rs31451122	Han Chinese (20635)	n/a	Tested on reference panels	Zhou et al. (2016)
rs3909184	Diverse (160)	31.8% sn, 80.4% sp	TaqMan SNP genotyping assay	He et al. (2015)
	Han Chinese (361)	n/a	Tested on reference panels	de Bakker et al. (2006)
rs2844682	Han Chinese (361)	n/a	Tested on reference panels	de Bakker et al. (2006)

Sn = sensitivity, sp = specificity, n/a = information that is not available within the publication. Underlined SNVs were selected for technical validation.

### Analytical Validation

The TaqMan method of SNV genotyping (Shen, Abdullah, and Wang, 2009) has been used in other studies on HLA haplotype-tagging SNVs (He et al., 2015; Fang et al., 2019) and was used in this study on a ViiA7 Real-Time PCR System (Thermo Fisher Scientific) according to the manufacturer’s protocol, with an increase in the number of PCR amplification cycles from 40 to 50 where necessary. One of the selected SNVs, rs17179220, had an available TaqMan assay (Thermo Fisher Scientific, 2021): assay ID C__33415939_10. Custom assays for the other three were designed (Table 3). For rs1061235 and rs10484555, sequence was obtained from the National Center for Biotechnology Information (NCBI) website and submitted *via* Thermo Fisher’s custom TaqMan SNP genotyping assay design tool. For rs144012689, design was more complex, owing to the other SNV two base pairs away (rs2596496), which has the potential to interfere with assay performance, as identified by Fang et al. (2019). We requested two assays for the rs144012689, one additionally matching the nucleotide base C at the nearby rs2596496, and one to cover the nucleotide base G at rs2596496.

**TABLE 3:** Sequences submitted for TaqMan SNP Genotyping Assays.

Haplotype	SNV	Assay ID	Sequence
HLA-A*31:01	rs17179220	C__33415939_10	CCACATGGAC CGTCCTGGAG AGGGA [G/A] CTCCACATTT GAGTTCCTGT TTCAT^a^
	rs1061235	ANNK49J	CCCCTCACTG TGATGGATAT GAATTTGTTC ATGAATATTT TTTTCTATAG TGTGAGACAG CTGCCTTGTG TGGGACTGAG AGGCAAGANT TGTTCCNGCC CTTCCCTTTG TGACTTNAAG [A/T] ACCCTGACTT TGTTTCTGCA AAGGCACCTG CATGTGTCTG TGTTCNTGTA GGCATAATGT GAGGAGGTGG GGAGACCACC CCACCCCCAT GTCCACCATG ACCCTCTTCC CACGCTGACC^b^
HLA-B*15:02	rs10484555	ANPRV67	CATCTAAAGT ATACAATTCA ATGGTTTTTC ACATATTCAG AGTTTTAAAT CTGCATATTT AATTTTAGAA CATTTTCATC ATCCCAAGAT AATCCACGGC TTTATAATAT GTCTCTCTAT [G/A] CAATGAAGTA AATCCATAAA TTTTGAGTTT TCACTTCAGG AGTGTTTTTT CTATCATTGG CAACTTTCCT AGCTTGACAG GCACCTTTCC AAGTACCTTT AACAATTTTA TTTTTAATAG^b^
	rs144012689	Submitted	CAAGCCCCAG GTAGAAGTGT TCCCTGCCTC ATTACTGGGA AGCAGCAT**(C/G)**C [A/T] CACAGGGGCT AANGCAGCCT GGGACCCTGT GTGCCAGCAC TTACTCTTTT
		AN2XMRN	TAGCCCCTGTG [T/A] G**C**ATGCTGCT^c^
		AN33GCK	TAGCCCCTGTG [T/A] G**G**ATGCTGCT^c^

a Sequence via Thermo Fisher website.

b Sequence from National Center for Biotechnology Information (NCBI) website, submitted to Thermo Fisher’s Custom TaqMan Assay Design Tool.

c Sequence via Thermo Fisher technical support, after discussion of the incorporation of rs2596496, indicated in red.

Samples were genotyped in duplicate, with an in-house automated method of conducting quality control on the data arising from the two technical replicates, and repeats being conducted as necessary. Data were analyzed using QuantStudio real-time PCR software, with manual adjustment of the genotyping result was conducted if required, based on the amplification data, in a manner that we have previously used (Carvalho Henriques et al., 2020a; Carvalho Henriques et al., 2020b). The software is unable to accurately assign calls when the number of clusters of samples in the allelic discrimination plots is greater than three or if there are too few numbers in specific clusters, so manual calls can ensure usable data can still be extracted.

### Samples

Twenty-nine samples with haplotypes identified by the 1000 Genomes Project (The 1000 Genomes Project Consortium et al., 2015) or the GeT-RM Collaborative Project were purchased from the Coriell Institute for Medical Research. The 1000 Genomes Project determined haplotypes via whole genome sequencing, targeted exome sequencing, and high-density single nucleotide polymorphism (SNP) microarrays. The GeT-RM Collaborative project determined haplotypes *via* next generation sequencing and compared results to those from sequence-specific oligonucleotide and sequence-specific priming technologies. A further nine samples had haplotypes catalogued in the UCLA International HLA DNA Exchange. This resource determined haplotypes *via* cross-reference between multiple labs globally, which employed various techniques including sequence-specific oligonucleotide technology and next generation sequencing. All samples had either known ancestral background, race, or region of sample acquisition.

## Results

### Tag SNV Selection

Rs10484555 was identified as a tag SNV for HLA-B*15:02 by two recent studies, one of them being Erlichster et al., described above. They found rs10484555 to have 100% sensitivity and 98.7% specificity for tagging HLA-B*15:02. Liu et al. (2015) identified tag SNVs by sequencing in 880 Han Chinese samples, confirmed the results in 500 additional samples, and reported rs10484555 to have 100% sensitivity and 99.3% specificity for tagging HLA-B*15:02 in this ancestral group.

Rs144012689 was identified as a tag SNV for HLA-B*15:02 by two recent studies. Gui et al. (2018) used sequencing data from 184 Han Chinese samples and confirmed their findings in a reference panel of 10,986 of the same ancestry. They found rs144012689 to have 96.7% sensitivity and 99.3% specificity for tagging HLA-B*15:02 in Han Chinese. Fang et al. (2019) identified rs144012689 as a potential tag for HLA-B*15:02, and tested association in 28,897 individuals in the United States, reporting 100% sensitivity and 99.97% specificity. The specificity was not 100% owing to the presence of rs144012689 in the HLA-B*15:13 haplotype, which is found at approximately 1/10 the frequency of HLA-B*15:02 (only eight identified in the sample of 28,897). This study also designed a TaqMan custom SNP Genotyping Assay for rs144012689, which they found to be 100% concordant with their sequencing results.

Rs31451122, rs3909184, and rs2844682 were also identified from literature as possible tag SNVs for HLA-B*15:02, but were not selected for further analysis. Rs31451122 was identified by a large-scale sequencing study (Zhou, et al., 2016), though samples were only from the Han Chinese population. Rs3909184 was identified by two studies, though one found this SNV to only have a sensitivity of 31.8% and a specificity of 80.4% for the tagging of HLA-B*15:02 (He et al., 2015). Rs2844682 was identified solely in the Han Chinese population, and specific linkage disequilibrium results or tagging ability was not provided (de Bakker et al., 2006).

Rs17179220 was identified by two recent studies as a tag SNV for HLA-A*31:01 (Erlichster et al., 2019; Zhou, et al., 2016). The study by Erlichster et al. used five large HLA reference panels, comprising 16,749 total samples, for tag SNV discovery and then validated all identified SNVs on a set of 955 ancestrally diverse samples from the 1000 Genomes dataset. Their aim was to identify tags that could function across multiple ethnicities; their results of rs17179220 having 100% sensitivity, and 99.6% specificity for tagging HLA-A*31:01 take into account African, admixed American, East Asian, and European populations. The study by Zhou et al. sequenced the entire HLA region in 20,635 individuals of Han Chinese ancestry and found a linkage disequilibrium r^2^ value of 0.972 between rs17179220 and the HLA-A*31:01 haplotype.

Rs1061235 was identified as a tag SNV for HLA-A*31:01 by three studies. de Bakker et al. (2006) used 361 samples of diverse ancestry from the International HapMap project for tag SNV discovery *via* PCR sequence-specific oligonucleotide priming (PCR-SSOP), and tested transferability on 996 other samples. Across all ancestral groups in which rs1061235 was identified, the r^2^ value was 1.000. Thorstensen et al. (2014) used a polymerase chain reaction-restriction fragment length polymorphism assay to investigate rs1061235 as a tag for HLA-A*31:01 in Norwegians. In a set of 204 samples, their assay had 100% sensitivity and 99.5% specificity for tagging HLA-A*31:01. The specificity was not 100% since rs1061235 may also be found on HLA-A*33:01 and HLA-A*33:03. Given the lower frequency of the HLA-A*33 haplotypes as compared to HLA*31:01 in Norwegians, the authors did not view this a major concern. However, these variants are more frequent in North America and the authors suggested that more work would have to be done before adopting rs1061235 as a tag SNV for HLA-A*31:01 in a multi-ethnic population such as Canada. The most recent study (He et al., 2015) tested 160 samples of diverse ancestry using a custom-designed rs1061235 TaqMan SNP Genotyping assay. They found the sensitivity to be 100% and the specificity to be 84% for tagging HLA-A*31:01.

Rs2571375, rs1150738, rs3869066, rs259945, and rs41541222 were also identified from literature as possible tag SNVs for HLA-A*31:01, but were not selected for further analysis. This was primarily due to a lack of data on these SNVs outside of select Asian ancestral groups. Rs2571375 was identified by only one study that was conducted in a Han Chinese population (Gui et al., 2018). Rs1150738, rs3869066, and rs259945 were all identified in one study in a Japanese population (Maekawa et al., 2015). Rs41541222 was identified by one study in an Asian population in 2012, but has not since been reported in any more recent literature (Aoki et al., 2012).

### TaqMan Assay Analytical Validation

**FIGURE 1:**
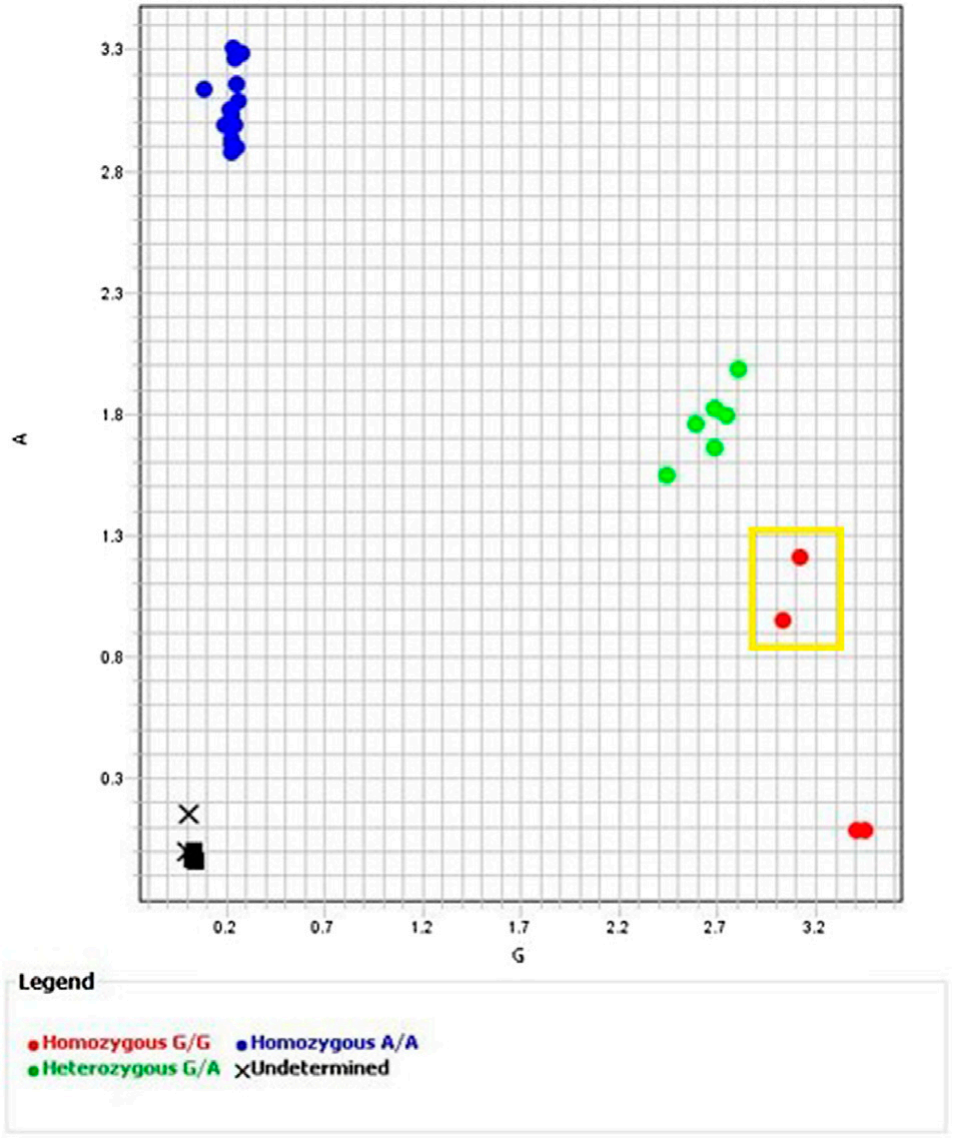
Allelic Discrimination Plot for ANPRV67 (rs10484555) Black “X”s represent sample 755, which failed to amplify. Axes indicate relative fluorescence of VIC (*x*-axis) and FAM (*y*-axis) dyes. Sample NA17019 surrounded by the yellow box. Though software assigned a homozygous G/G call, the position on the allelic discrimination plot and multicomponent plot (Figure 2 **(A)**) clearly indicate a heterozygous G/A call.

**FIGURE 2:**
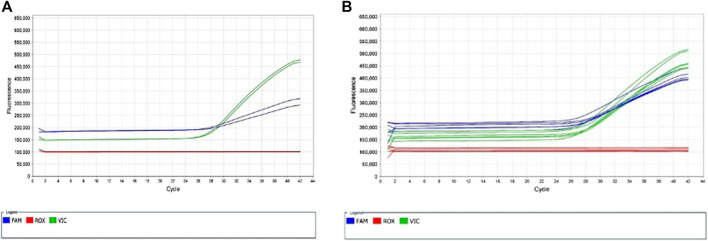
Multicomponent Plots for ANPRV67 (rs10484555). **(A)** Fluorescence of both VIC and FAM dyes indicate a heterozygous call for sample NA17019. A homozygous G/G sample would show solely fluorescence of the VIC dye. **(B)** Fluorescence of both VIC and FAM dyes from remainder of heterozygous samples (green circles in Figure 1, samples 738, HG00463, NA23090).

Table 4 summarizes the results for each assay. The assay targeting rs10484555, ANPRV67, identified HLA-B*15:02 with 100% sensitivity and 100% specificity (Figure 1). There were no false positives or false negatives, but sample 755 failed to amplify on multiple runs, so no result could be obtained. If included, the sensitivity and specificity of the assay would both be reduced to 97%. The sample worked well on other assays, so it is likely that nearby SNVs, insertions, or deletions interfered with the ability of the probe to bind to that sample. Sample NA17019 required manual calling owing to weak amplification of the FAM dye (Figure 2).

**TABLE 4:** Reference sample prior haplotype data and TaqMan assay results.

Sample	*HLA-A*	*HLA-B*	Available population information	C__33415939_10 (rs17179220	ANNK49J (rs1061235)	ANPRV67 (rs10484555)	AN33GCK (rs144012689, rs2596496G)
HG00096	A*01:01/A*29:02	B*08:01/B*44:03	England/Scotland	G/G	A/A	A/A	T/T
HG00463	A*02:07/A*11:01	B*15:02/B*46:01	Han Chinese in Beijing	G/G	A/A	G/A	A/T
HG01190	A*02:01/A*24:02	B*18:01/B*15:20	Puerto Rico	G/G	A/A	A/A	T/T
HG01791	A*01:01/A*25:01	B*07:02/B*08:01	England/Scotland	G/G	A/A	A/A	T/T
NA09301	A*23:01/A*30:01	B*13:02/B*49:01	Ashkenazi	G/G	A/A	A/A	T/T
NA10005	A*02:01/A*33:03	B*15:03/B*44:02	Black in United States	G/G	A/T	A/A	T/T
NA10860	A*02:01/A*29:02	B*35:01/B*44:03	Utah Mormon	G/G	A/A	A/A	T/T
NA12244	A*02:01/A*23:01	B*15:01/B*41:01	Utah Mormon	G/G	A/A	A/A	T/T
NA17019	A*02:01/A*11:01	B*15:02/B*15:11	Chinese	G/G	A/A	G/A^a^	T/T
NA17039	A*30:01/A*31:01	B*35:08/B*42:02	Black in United States	G/A	A/T	A/A	T/T
NA17213	A*02:01/A*24:02	B*27:05/B*51:01	European	G/G	A/A	A/A	T/T
NA17218	A*01:01/A*01:01	B*07:02/B*08:01	European	G/G	A/A	A/A	T/T
NA17227	A*01:01/A*02:01	B*08:01/B*40:02	Welsh	G/G	A/A	A/A	T/T
NA17229	A*24:02/A*31:01	B*35:01/B*35:20	European	G/A	A/T	A/A	T/T
NA17235	A*03:01/A*31:01	B*07:02/B*40:01	European	G/A	A/T	A/A	T/T
NA17243	A*24:02/A*29:02	B*40:01/B*44:03	European	G/G	A/A	A/A	T/T
NA17244	A*02:01/A*30:02	B*13:02/B*51:01	European	G/G	A/A	A/A	T/T
NA17256	A*01:01/A*30:01	B*13:02/B*40:01	European	G/G	A/A	A/A	T/T
NA17261	A*01:01/A*03:01	B*35:08/B*44:02	European	G/G	A/A	A/A	T/T
NA17277	A*24:02/A*31:01	B*27:05/B*56:01	European	G/A	A/T	A/A	T/T
NA17281	A*02:01/A*31:01	B*39:06/B*56:01	European	G/A	A/T	A/A	T/T
NA17287	A*03:01/A*03:02	B*15:01/B*35:03	European	G/G	A/A	A/A	T/T
NA17292	A*02:01/A*02:01	B*07:02/B*51:01	European	G/G	A/A	A/A	T/T
NA17300	A*03:01/A*03:01	B*07:02/B*53:01	European	G/G	A/A	A/A	T/T
NA18545	A*11:01/A*24:02	B*40:01/B*40:01	Han Chinese in Beijing	G/G	A/A	A/A	T/T
NA18563	A*02:01/A*33:03	B*48:01/B*51:01	Han Chinese in Beijing	G/G	A/T	A/A	T/T
NA18565	A*11:01/A*24:02	B*15:25/B*51:01	Han Chinese in Beijing	G/G	A/A	A/A	T/T
NA23090	A*11:01/A*11:01	B*15:02/B*51:01	United States	G/G	A/A	G/A	A/T
NA23093	A*11:01/A*11:01	B*15:02/B*15:02	Chinese	G/G	A/A	G/G	A/A
689	A*31:01/BLANK ^*b*^	B*39:01/B*39:11	Hispanic	A/A	T/T	A/A	T/T
691	A*31:01/A*02:01	B*14:02/B*48:01	Hispanic	G/A	A/T	A/A	T/T
738	A*11:02/A*26:01	B*15:02/B*27:04	Filipino	G/G	A/A	G/A	A/T
740	A*31:01/A*24:02	B*38:01/B*51:01	European	A/G	A/T	A/A	T/T
750	A*31:01/A*33:03	B*40:02/B*58:01	Hispanic	A/G	T/T	A/A	T/T
754	A*31:01/A*68:01	B*15:15/B*39:06	Hispanic	A/G	A/T	A/A	T/T
755	A*31:01/A*24:02	B*39:02/B*39:06	Hispanic	A/G	A/T	fail^c^	T/T
757	A*31:01/A*23:01	B*15:10/B*35:17	Hispanic	A/G	A/T	A/A	T/T
810	A*31:01/A*02:01	B*51:02/B*56:01	Hispanic	A/G	A/T	A/A	T/T

^a^Manual call, software automatically called G/G, though G/A correct due to amplification of both VIC and FAM dyes.

^b^Sample only containing one haplotype, other blank for unknown reason, potentially gene deletion, results in homozygous TaqMan calls for *HLA-A*.

^c^Sample failed to show any amplification on multiple runs of assay ANPRV67.

**FIGURE 3:**
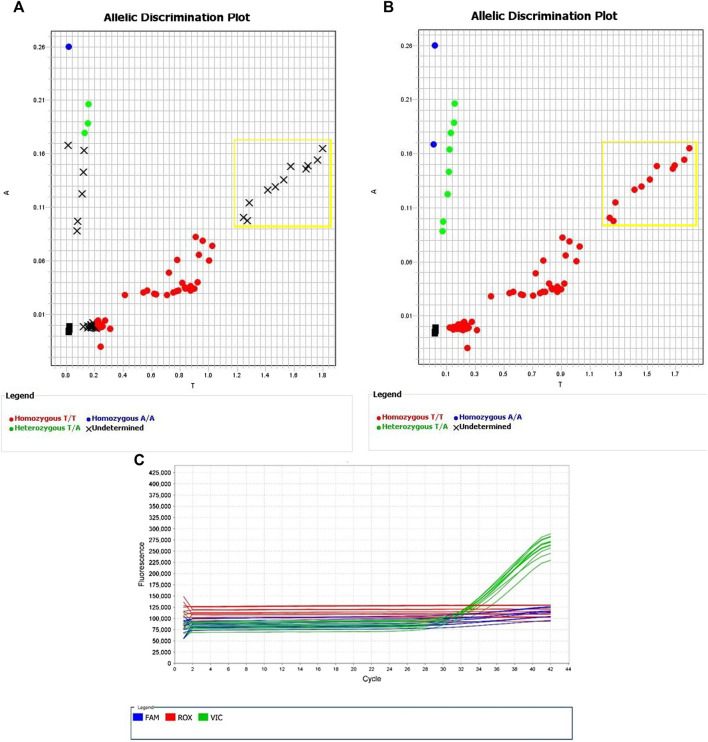
Allelic Discrimination Plots and Multicomponent Plot for AN2XMRN (rs144012689, rs2596496C)Samples in the yellow box showed most significant amplification (NA17235, NA17256, NA18545, 689, 691, 755), in comparison to the rest of the samples. This group likely has the nucleotide base C at rs2596496. **(A)** shows the software-generated calls, and **(B)** shows manually assigned calls based on relative fluorescence. **(C)** Fluorescence of VIC dye on multicomponent plot for NA17235, NA17256, NA18545, 689, 691, 755 (samples in yellow box) indicates homozygous T/T call.

**FIGURE 4:**
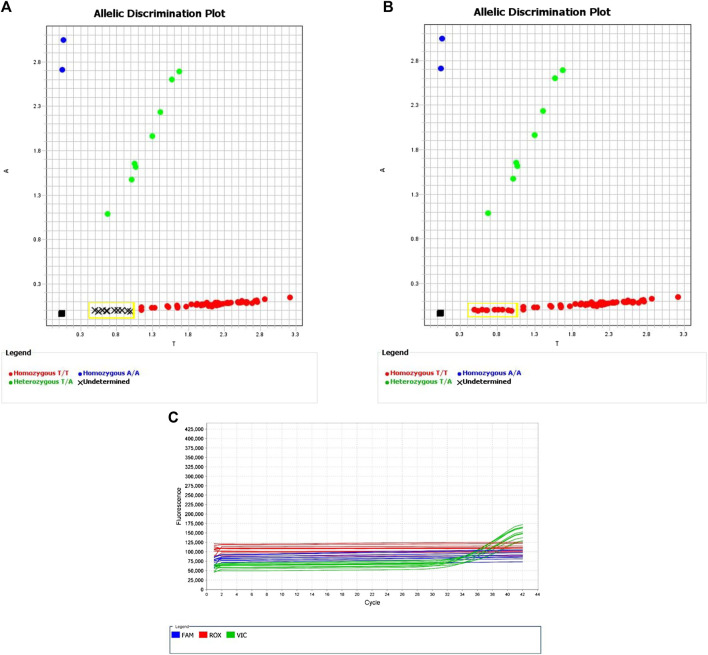
Allelic Discrimination Plots and Multicomponent Plot for AN33GCK (rs144012689, rs2596496G)Black “X”s indicate samples for which the automatically generated calls failed. Samples in yellow box are the ones that showed the most significant amplification on assay AN2XMRN (NA17235, NA17256, NA18545, 689, 691, 755). **(A)** shows the software-generated calls, and **(B)** shows manually assigned calls based on relative fluorescence. **(C)** Fluorescence of VIC dye on multicomponent plot for NA17235, NA17256, NA18545, 689, 691, 755 (samples in yellow box) indicates homozygous T/T call.

The assay targeting rs144012689 with the nucleotide base C at rs2596496, AN2XMRN, showed poor amplification for the majority of samples (Figure 3), with the exception of one group of samples showing strong amplification of the VIC dye (NA17235, NA17256, NA18545, 689, 691, 755). The assay targeting rs144012689 with the nucleotide base G at rs2596496, AN33GCK, showed strong amplification of most samples (Figure 4), with the exception of the same group as above. As these samples amplified well with assay AN2XMRN, we inferred that they have the C nucleotide at rs2596496. Manual calling of the group of six samples with assay AN33GCK (Figure 4) resulted in 100% sensitivity and specificity for the identification of HLA-B*15:02.

The assay targeting rs17179220, C__33415939_10, identified HLA-A*31:01 with 100% sensitivity and 100% specificity. Using the default genotyping settings in the Quant Studio software, amplification occurred late in the reaction and sample discrimination was challenging; however, increasing the number of PCR amplification cycles from 40 to 50 made the results much clearer.

The assay targeting rs1061235, ANNK49J, identified HLA-A*31:01 with 100% sensitivity and 95% specificity. All three false positives were due to the presence of rs1061235 on the haplotype HLA-A*33:03.

## Discussion

Our data are consistent with rs10484555 and rs17179220 being the best tagging SNVs for HLA-B*15:02 and HLA-A*31:01 respectively, across diverse ancestral backgrounds.

Assay ANPRV67 for rs10484555 had 100% sensitivity and specificity for HLA-B*15:02 (excluding one sample, 755, that failed to amplify on multiple attempts). We suggest further investigation, such as sequencing, of sample 755 and one sample that required manual calling (NA17019) is warranted. Our data are consistent with the database prediction of Erlichster et al. (2019) of 100% sensitivity and 98.7% specificity of rs10484555 for tagging HLA-B*15:02 in a diverse population, and with the 100% sensitivity and 99.3% specificity for tagging HLA-B*15:02 in the Han Chinese population reported by Liu et al. (2015).

Assay AN33GCK for rs144012689 with the nucleotide base G at rs2596496, in combination with manual calling, resulted in 100% sensitivity and 100% specificity for the identification of HLA-B*15:02. However, if another nucleotide other than C or G is present at rs2596496, which is theoretically possible (National Center for Biotechnology Information, 2020), the assay may not work. Even though there are no frequency data available for the other possible nucleotide variants, we suggest further custom assays for the other nucleotide possibilities at rs2596496 be developed to rule out this possibility. The presence of the C or G nucleotide within our sample set is not population-specific, as the aforementioned group of samples we hypothesize to have the C nucleotide at rs2596496 are from European, Han Chinese, and Hispanic populations. As well, there is the potential for false positives in individuals with the HLA-B*15:13 haplotype. This haplotype has a global frequency of 0.08%, in comparison to a frequency of 0.91% for HLA-B*15:02 (Fang et al., 2019), which is small but not negligible. Overall, for an HLA-B*15:02 haplotyping strategy, we suggest using assay ANPRV67 for rs10484555 first, and if any output such as that seen with samples 755 and NA17019 results, assay AN33GCK for rs144012689 may be additionally used.

Assay C__33415939_10 for rs17179220 had 100% sensitivity and specificity for HLA-A*31:01. This is comparable to the database prediction of Erlichster et al. (2019) of 100% sensitivity and 99.6% specificity of rs17179220 for tagging HLA-A*31:01. The assay performs best when the number of PCR amplification cycles is increased to 50 (increasing total run time from 90 to 110 min). Assay ANNK49J for rs1061235 also identified HLA-A*31:01 with 100% sensitivity, with its specificity impacted by this SNV also being present in the HLA-A*33:01 and HLA-A*33:03 haplotypes (Thorstensen et al., 2014). A study in an ancestrally-diverse group of Canadian children (Amstutz et al., 2013) found that out of 20 children carrying the minor allele of rs1061235, 12 had a haplotype of HLA-A*31:01, five had a haplotype of HLA-A*33:03, and three had a haplotype of HLA-A*33:01. As a result, they concluded that rs1061235 is not an optimal tag for HLA-A*31:01 in ancestrally diverse populations. However, they did find that the rs1061235 minor allele was overrepresented among carbamazepine hypersensitivity cases in patients not carrying the HLA-A*31:01 haplotype, suggesting a possible association of the HLA-A*33 haplotypes with such hypersensitivity. Further research regarding this is required.

The selection of positive controls, especially for minor alleles that are less represented within a sample set, has the potential to improve clustering and the ability of the QuantStudio software to make accurate automatic calls. For this purpose, we recommend NA23093 as a homozygous HLA-B*15:02 control and HG00463 or NA23090 as a heterozygous HLA-B*15:02 control. Numerous Coriell samples would be suitable for heterozygous HLA-A*31:01 controls (NA17039, NA17229, NA17235, NA17277, NA17281). A limitation of our study is that owing to the limited availability of reference samples with relevant haplotypes, our analysis was limited to only 38 samples, primarily of European ancestry, with Hispanic, Han Chinese, Filipino, and Black American representation. It has been previously noted that population sets that include larger numbers of those of different ancestry may see higher rates of false positives (Liu et al., 2015). Nonetheless, as the ADRs associated with these haplotypes on prescribing carbamazepine or oxcarbazepine may be fatal, individuals may not know the full details of their ancestry, and certainly testing HLA-B*15:02 is indicated in individuals had physician-reported ethnicity other than Asian (Fang et al., 2019) we recommend using these SNVs to identify these haplotypes in individuals of any ancestral group. Another potential limitation in the translation of this work to the clinical laboratory setting is the need for occasional manual calls. This is in fact not unusual with this and other techniques in this setting: data would need to be reviewed before exporting files or reporting to patients. This process is not particularly lengthy or challenging but does require some knowledge of the TaqMan assays and use of the QuantStudio software. Different population background and sample quality may be relevant to the amount of data that requires manual manipulation. As mentioned, the proximity of nearby SNVs to the SNVs of interest can interfere with assay performance, and certain SNVs may be more prevalent in certain ancestral groups. Poor sample quality or low concentration may also increase the prevalence of data that requires manual calls.

## Conclusion

In summary, we have validated a method of detection of HLA-B*15:02 and HLA-A*31:01 that at <$1USD per sample plus labour of less than 2 h is orders of magnitude lower than the cost of haplotyping by the current “gold-standard” methods. A recent study that aimed to assess the cost-effectiveness of pre-emptive pharmacogenomic testing for HLA haplotypes, based on their relative frequencies worldwide, concluded that for carbamazepine, pre-emptive genotyping of HLA-B*15:02 would be cost-effective across most of East and South Asia, whereas HLA-A*31:01 testing would be likely to be cost-effective globally (Zhou et al., 2021). However, their calculations assumed the cost per sample to be $40, which is much greater than our estimated cost of TaqMan SNP genotyping.

Our findings are relevant for other technologies that rely on SNV detection for the identification of HLA haplotypes. For example, the Ion AmpliSeq Pharmacogenomics Panel uses rs1061235 to tag HLA-A*31:01. Although we and other investigators have found that the specificity of this SNV as a haplotype tagging marker is not 100%, as carbamazepine hypersensitivity has been found in patients with the rs1061235 minor allele but not carrying the HLA-A*31:01 haplotype (Amstutz et al., 2013), the use of this SNV to screen for carbamazepine hypersensitivity should be adequate. Further, the sensitivity is 100% in our data and that of others, and given the severity of the potential ADRs, the absence of false negatives is more important than the absence of false positives. Agena Biosciences use markers including rs10484555 for HLA-B*15:02 and rs1061235 for HLA-A*31:01, which should be fine. Pharmacoscan includes rs1061235 as a screen for HLA-A*31:01 as well. Cross-validation of our method could be undertaken versus the multiple technologies above described in a manner that we have previously described (Carvalho Henriques, et al., 2020a; 2020c).

## Data Availability

The datasets presented in this study can be found in online repositories. The names of the repository/repositories and accession number(s) can be found below: https://figshare.com/articles/dataset/EDS_files_raw_data_from_QuantStudio_for_Validation_of_Single_Nucleotide_Variant_Assays_for_Human_Leukocyte_Antigen_Haplotypes_HLA-B_15_02_and_HLA-A_31_01_Across_Diverse_Ancestral_Backgrounds/14977929.
