# Corrigendum: Validation of single nucleotide variant assays for human leukocyte antigen haplotypes HLA-B*15:02 and HLA-A*31:01 across diverse ancestral backgrounds

**DOI:** 10.3389/fphar.2022.971316

**Published:** 2022-08-05

**Authors:** Amanda Buchner, Xiuying Hu, Katherine J. Aitchison

**Affiliations:** ^1^ Department of Psychiatry, Faculty of Medicine and Dentistry, University of Alberta, Edmonton, AB, Canada; ^2^ Neuroscience and Mental Health Institute, University of Alberta, Edmonton, AB, Canada; ^3^ Department of Medical Genetics, Faculty of Medicine and Dentistry, University of Alberta, Edmonton, AB, Canada

**Keywords:** HLA antigens, adverse drug reactions, carbamazepine, psychiatry, pharmacogenetics, precision medicine, single nucleotide variants, oxcarbazepine

Text Correction.

In the published article, there was an error. Several “rs” numbers were written incorrectly.

A correction has been made to **Abstract**. This sentence previously stated:

“A custom assay for rs106235 identified HLA-A*31:01 with 100% sensitivity and 95% specificity. The slight reduction in specificity for the latter was owing to another haplotype (HLA-A*33:03) also being detected. While any positive call using the rs106235 assay could therefore be further investigated, as the presence of the HLA-A*31:01 haplotype confers adverse drug reaction risk, the absence of false negatives (indexed by sensitivity) is more important than false positives.”

The corrected sentence appears below:

“A custom assay for rs1061235 identified HLA-A*31:01 with 100% sensitivity and 95% specificity. The slight reduction in specificity for the latter was owing to another haplotype (HLA-A*33:03) also being detected. While any positive call using the rs1061235 assay could therefore be further investigated, as the presence of the HLA-A*31:01 haplotype confers adverse drug reaction risk, the absence of false negatives (indexed by sensitivity) is more important than false positives.”

A correction has been made to **Results**, TaqMan Assay Analytical Validation, **Paragraph 3**. This sentence previously stated:

“The assay targeting rs17179220, C__33414939_10, identified HLA-A*31:01 with 100% sensitivity and 100% specificity.”

The corrected sentence appears below:

“The assay targeting rs17179220, C__33415939_10, identified HLA-A*31:01 with 100% sensitivity and 100% specificity.”

A correction has been made to **Discussion**, **Paragraph 4**. This sentence previously stated:

“Assay C__33414939_10 for rs17179220 had 100% sensitivity and specificity for HLA-A*31:01.”

The corrected sentence appears below:

“Assay C__33415939_10 for rs17179220 had 100% sensitivity and specificity for HLA-A*31:01.”

The authors apologize for this error and state that this does not change the scientific conclusions of the article in any way. The original article has been updated.

